# Out-of-Pocket Costs for SGLT-2 (Sodium-Glucose Transport Protein-2) Inhibitors in the United States

**DOI:** 10.1161/CIRCHEARTFAILURE.121.009099

**Published:** 2021-12-10

**Authors:** Rahul Aggarwal, Muthiah Vaduganathan, Nicholas Chiu, Deepak L. Bhatt

**Affiliations:** Department of Medicine, Beth Israel Deaconess Medical Center, Harvard Medical School, Boston, MA (R.A., N.C.).; Brigham and Women’s Hospital Heart and Vascular Center, Harvard Medical School, Boston, MA (M.V., D.L.B.).

**Keywords:** heart disease, insurance, kidney, Medicare, pharmacy

SGLT-2 (sodium-glucose transport protein-2) inhibitors are indicated for millions of US individuals with heart disease, diabetes, or kidney dysfunction.^1,2^ However, these medications have a high retail price, at over $500 per month ($16 per pill).^3^ The high costs may contribute to physician inertia to prescribe therapy, impede early initiation, and decrease patient adherence.^3,4^

Because of these effects, understanding the out-of-pocket costs for SGLT-2 inhibitors is essential. However, estimates for out-of-pocket costs are limited with no nationally representative or multi-payer estimates.^3^ We aim to address this gap in evidence by providing national estimates of out-of-pocket costs for SGLT-2 inhibitors, stratifying results by major insurance payor types.

The Medical Expenditure Panel Survey (MEPS; 2014–2018) is administered to a nationally representative sample of civilian-noninstitutionalized persons in the United States and is designed to estimate cost measures for prescription medications in the United States.^5^ MEPS collects prescription total expenditures and out-of-pocket costs through pharmacy payment records.

Outcomes were mean monthly total expenditures per person for SGLT-2 inhibitors and out-of-pocket costs. Results were stratified by insurance type (private, Medicare, Medicaid, dual Medicare/Medicaid, Medicare with supplemental private, and other insurances). Medicare categories only included adults ≥65 years of age, whereas private and only Medicaid categories included only adults <65 years of age. National projections were estimated using MEPS survey weights by accounting for the complex multistage design and participant nonresponse rates, with CIs computed using the Taylor-series linearization method. Subgroup estimations were determined by use of statistical software accounting for the survey design. Costs were inflation-adjusted to the 2018 Consumer Price Index for prescription medications. Comparisons among different insurance payors were assessed using linear regression, with Medicare-insured adults as the comparator group. The data used in this study is publicly available from the Agency for Healthcare Research and Quality. Analytic methods as well as study materials are available to other researchers on request to the authors for purposes of reproducing the results or replicating the procedure. MEPS is Institutional Review Board approved by the Westat Institutional Review Board.

Of 167 298 individuals in MEPS, we identified 504 adults prescribed SGLT-2 inhibitors. After applying survey weights to these 504 adults, we estimated that in the United States, 1 133 139 (95% CI, 958 771–1 307 507) adults were on SGLT-2 inhibitors. Annually, an estimated 5 636 828 (95% CI, 4 619 082–6 654 574) prescriptions for SGLT-2 inhibitors were filled, yielding estimated national total expenditures of $3 308 539 528 (95% CI, $2 722 122 074–$3 894 956 982). Of these individuals, 58.0% had private insurances, 9.9% had Medicare, 7.3% had Medicaid only, 4.4% had dual Medicare/Medicaid, 14.4% had Medicare with private supplemental insurance, 2.4% had other insurances, and 3.6% had no insurance.

Mean total expenditure for SGLT-2 therapy was $416.12 (95% CI, $405.98–$426.27) per person per month, with an average monthly out-of-pocket expense of $46.70 (95% CI, $36.76–$56.64) per person. Compared with Medicare-insured individuals ($450.15 [95% CI, $424.18–$476.11]), total monthly per person costs were less for privately insured individuals ($413.42 [95% CI, $400.09–$426.74], difference −$36.73, *P*=0.020) and uninsured individuals ($300.69 [95% CI, $201.96–$399.42], difference −$149.46, *P*=0.005), but similar for the other insurance groups (Table). Out-of-pocket expenses, when compared with Medicare-insured individuals ($49.42 [95% CI, $25.41–$73.43]) were lowest for Medicaid ($5.10 [95% CI, $0.34–$9.86], difference −$44.32, *P*=0.001) and dual Medicare/Medicaid insured adults ($3.87 [95% CI, $1.00–$6.75], difference −$45.54, *P*=0.001) but similar for the other insurance groups (Table).

**Table 1.**
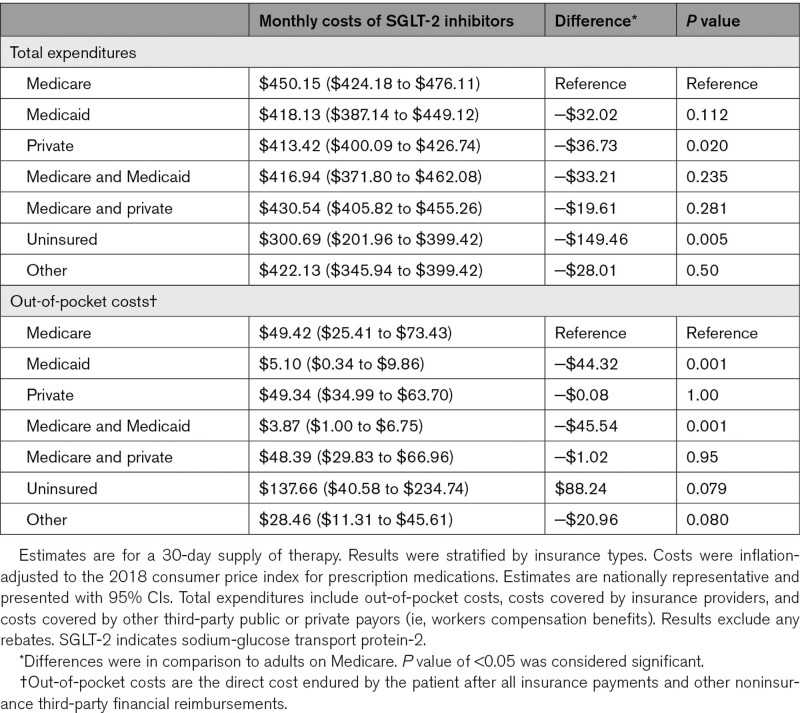
Total Expenditures and Out-of-Pocket Costs for SGLT-2 Inhibitors

Total expenditures for SGLT-2 inhibitors were high—at over $400 per month. However, patients were protected from most of these costs, with average out-of-pocket spending at $47 per month (12% of total expenditures). Out-of-pocket costs were lowest for those covered by Medicaid ($4−$5 per month).

Our findings suggest that insurance coverage for therapy was generally substantial. However, out-of-pocket costs for uninsured individuals was high with an estimated average cost of $138 per month, or $5 per day. Strategies to mitigate the high costs for this group of patients, who are already predisposed to having financial hardship, are necessary. Further, patients with Medicare or private insurances still endured ≈$50 per month in costs, which may be prohibitive for some, especially patients on multiple expensive medications. Private insurances were found to have lower total per person expenditures for SGLT-2 inhibitors than Medicare, which may reflect better price negotiation by private insurances. While a prior report estimated higher out-of-pocket costs for Medicare beneficiaries, our study included low-income adults eligible for subsidies that were excluded in the prior report.^3^

Limitations include inability to account for health insurance premiums, deductibles, or rebates. Health insurance coverage and plans may have dynamically changed during the study, and we only provide a cross-sectional estimate. We could only identify individuals who received therapy, resulting in exclusion of individuals who found costs for therapy prohibitive. Additionally, sample size may limit the precision of our estimates.

In conclusion, in the United States, the majority of total expenditures for SGLT-2 inhibitors were not endured by patients, though substantial variation for coverage existed by insurance type. Certain patients may have difficulty affording therapy due to these differences in out-of-pocket costs, especially adults without insurance.

## Article Information

### Sources of Funding

None.

### Disclosures

Dr Bhatt discloses the following relationships—Advisory Board: Cardax, CellProthera, Cereno Scientific, Elsevier Practice Update Cardiology, Level Ex, Medscape Cardiology, MyoKardia, PhaseBio, PLx Pharma, Regado Biosciences; Board of Directors: Boston VA Research Institute, Society of Cardiovascular Patient Care, TobeSoft; Chair: American Heart Association Quality Oversight Committee; Data Monitoring Committees: Baim Institute for Clinical Research (formerly Harvard Clinical Research Institute, for the PORTICO trial, funded by St. Jude Medical, now Abbott), Cleveland Clinic (including for the ExCEED trial, funded by Edwards), Contego Medical (Chair, PERFORMANCE 2), Duke Clinical Research Institute, Mayo Clinic, Mount Sinai School of Medicine (for the ENVISAGE trial, funded by Daiichi Sankyo), Population Health Research Institute; Honoraria: American College of Cardiology (Senior Associate Editor, Clinical Trials and News, ACC.org; Vice-Chair, ACC Accreditation Committee), Baim Institute for Clinical Research (formerly Harvard Clinical Research Institute; RE-DUAL PCI clinical trial steering committee funded by Boehringer Ingelheim; AEGIS-II executive committee funded by CSL Behring), Belvoir Publications (Editor in Chief, Harvard Heart Letter), Canadian Medical and Surgical Knowledge Translation Research Group (clinical trial steering committees), Duke Clinical Research Institute (clinical trial steering committees, including for the PRONOUNCE trial, funded by Ferring Pharmaceuticals), HMP Global (Editor in Chief, Journal of Invasive Cardiology), Journal of the American College of Cardiology (Guest Editor; Associate Editor), K2P (Co-Chair, interdisciplinary curriculum), Level Ex, Medtelligence/ReachMD (CME steering committees), MJH Life Sciences, Population Health Research Institute (for the COMPASS operations committee, publications committee, steering committee, and USA national co-leader, funded by Bayer), Slack Publications (Chief Medical Editor, Cardiology Today’s Intervention), Society of Cardiovascular Patient Care (Secretary/Treasurer), WebMD (CME steering committees); Other: Clinical Cardiology (Deputy Editor), NCDR-ACTION Registry (National Cardiovascular Data Registry, Action Registry) Steering Committee (Chair), VA CART (VA Cardiovascular Assessment, Reporting, and Tracking) Research and Publications Committee (Chair); Research Funding: Abbott, Afimmune, Amarin, Amgen, AstraZeneca, Bayer, Boehringer Ingelheim, Bristol-Myers Squibb, Cardax, Chiesi, CSL Behring, Eisai, Ethicon, Ferring Pharmaceuticals, Forest Laboratories, Fractyl, HLS Therapeutics, Idorsia, Ironwood, Ischemix, Lexicon, Lilly, Medtronic, MyoKardia, Owkin, Pfizer, PhaseBio, PLx Pharma, Regeneron, Roche, Sanofi, Synaptic, The Medicines Company; Royalties: Elsevier (Editor, Cardiovascular Intervention: A Companion to Braunwald’s Heart Disease); Site Co-Investigator: Biotronik, Boston Scientific, CSI, St. Jude Medical (now Abbott), Svelte; Trustee: American College of Cardiology; Unfunded Research: FlowCo, Merck, Novo Nordisk, Takeda. Dr Vaduganathan has received research grant support or served on advisory boards for American Regent, Amgen, AstraZeneca, Bayer AG, Baxter Healthcare, Boehringer Ingelheim, Cytokinetics, Lexicon Pharmaceuticals, and Relypsa, speaker engagements with Novartis and Roche Diagnostics, and participates on clinical end point committees for studies sponsored by Galmed and Novartis. The other authors report no conflicts.
